# Optimizing spatio-temporal correlation structures for modeling food security in Africa: a simulation-based investigation

**DOI:** 10.1186/s12859-024-05791-w

**Published:** 2024-04-27

**Authors:** Adusei Bofa, Temesgen Zewotir

**Affiliations:** School of Mathematics, Statistics, and Computer Science, University of KwaZulu Natal, Oliver Tambo Building, Westville Campus, Durban, South Africa

**Keywords:** Bayesian poisson model, Markov chain monte carlo(MCMC), Matrix plot, Mean absolute error, Root mean square error, Watanabe akaike information criterion

## Abstract

This study investigates the impact of spatio- temporal correlation using four spatio-temporal models: Spatio-Temporal Poisson Linear Trend Model (SPLTM), Poisson Temporal Model (TMS), Spatio-Temporal Poisson Anova Model (SPAM), and Spatio-Temporal Poisson Separable Model (STSM) concerning food security and nutrition in Africa. Evaluating model goodness of fit using the Watanabe Akaike Information Criterion (WAIC) and assessing bias through root mean square error and mean absolute error values revealed a consistent monotonic pattern. SPLTM consistently demonstrates a propensity for overestimating food security, while TMS exhibits a diverse bias profile, shifting between overestimation and underestimation based on varying correlation settings. SPAM emerges as a beacon of reliability, showcasing minimal bias and WAIC across diverse scenarios, while STSM consistently underestimates food security, particularly in regions marked by low to moderate spatio-temporal correlation. SPAM consistently outperforms other models, making it a top choice for modeling food security and nutrition dynamics in Africa. This research highlights the impact of spatial and temporal correlations on food security and nutrition patterns and provides guidance for model selection and refinement. Researchers are encouraged to meticulously evaluate the biases and goodness of fit characteristics of models, ensuring their alignment with the specific attributes of their data and research goals. This knowledge empowers researchers to select models that offer reliability and consistency, enhancing the applicability of their findings.

## Introduction

Food security is a critical concern, particularly in the context of Africa, where numerous countries face persistent challenges in ensuring adequate access to safe, nutritious, and affordable food for their populations. Understanding the complex dynamics of food security is essential for policymakers, researchers, and practitioners to develop effective interventions and strategies to address this pressing issue.

Traditionally, food security analysis has focused on temporal aspects, examining trends and changes in food availability, access, and utilization over time [1]. Nicholson, Stephens [1] reiterated the overemphasis on availability and accessibility indicators. In their study, they put forth four recommendations: avoiding confusion between "food availability" and "food security," consolidating food access indicators, assessing stable outcomes for food security indicators, and establishing empirical data linking results from agricultural systems models to outcomes related to food access. This emphasizes the necessity of including a broader spectrum of variables when evaluating food security and nutrition, particularly within the African context. However, it is increasingly recognized that food security is not solely determined by temporal factors but is also influenced by spatial dynamics [2]. Cooper, Brown [3] conducted a review of factors associated with food security, analyzing 16,152 abstracts from 3297 publications spanning the years 1975 to 2018. The review revealed that a majority of the studied publications focused solely on spatial effects rather than considering spatio-temporal impacts. This highlights the necessity for exploring spatio-temporal modeling.

To capture spatial–temporal variations and better comprehend the intricacies of food security in Africa, there is a growing need for spatio-temporal modeling approaches. Spatio-temporal models integrate both the spatial and temporal dimensions of data, enabling a more comprehensive understanding of the underlying processes and patterns that shape food security dynamics [4]. Spatio-temporal modeling offers several advantages as it explores spatial dependencies, assesses temporal dependencies, and captures persistence, trends, and cyclical patterns in food security outcomes. Moreover, it provides a framework to investigate interactions between spatial and temporal factors, recognizing complex feedback loops and dynamics within food systems [5].

Over the years, Bayesian-based spatio-temporal modeling has emerged as a significant area of interest due to its versatile applications across various disciplines, including ecology and epidemiology [6]. Aswi, Cramb [7] conducted a substantive case study on Dengue Incidence, exploring a range of Bayesian spatio-temporal models and evaluating their goodness of fit using the Watanabe Akaike Information Criterion (WAIC). Similar research has also been conducted by Ibeji, Mwambi [8], Wahyuni and Syam [9], and Yoo and Wikle [10], showcasing the growing interest in Bayesian spatio-temporal modeling in different contexts.

Previous studies [1, 3] have employed spatio-temporal modeling from a frequentist perspective, driven by limitations such as the absence of subjectivity, challenges in handling prior information, and issues with non-robustness. Additionally, the difficulties in establishing efficient and reliable computing techniques for Intrinsic Conditional Autoregressive (ICAR) models, as outlined by Ver Hoef, Peterson [11], might have prompted the adoption of a spatio-temporal perspective, as seen in works by Fenta, Zewotir [12]and Sahu [13]. Are all spatio-temporal models robust to spatial and temporal correlations[14]? Hence, our goal was to conduct a comparative investigation into the impact of spatio-temporal correlation on four models [13] by evaluating their goodness of fit and biases in modeling food security in Africa. The Bayesian framework allows us to incorporate subjective insights, dealing adeptly with prior information and ensuring robustness—a notable improvement over the limitations identified in frequentist methodologies [11]

To assess goodness of fit, we employed the Watanabe-Akaike Information Criterion (WAIC) for model comparison. This criterion, based on the entire posterior distribution, strikes a balance between model fit and complexity, addressing concerns of both underfitting and overfitting. WAIC is specifically designed to estimate out-of-sample predictive accuracy, making it a versatile and valuable tool for researchers working with various types of Bayesian models [14]. Furthermore, we examined the extent of bias within this category of models through the utilization of Root Mean Square Error (RMSE) and Mean Absolute Error (MAE) measurements using Monte Carlo Simulations.

This comparison helps to understand how each model performs and whether certain models are more robust to variations in the spatio-temporal correlations. By achieving these objectives, the study sought to provide valuable insights into the suitability and performance of various spatio-temporal models in the context of food security analysis, especially considering the impact of different spatio-temporal correlations. The findings would contribute to a better understanding of the strengths and limitations of different modeling approaches and aid in making informed decisions when analyzing food security data in Africa.

Our methodology uniquely combines dimensionality reduction through PCA, Bayesian modeling, and simulation-based exploration, addressing the complexities of spatio-temporal correlation structures in the context of food security. This enables the exploration of diverse policy scenarios, ranging from weak to strong spatio-temporal correlation structures. By simulating these scenarios, we not only account for uncertainties but also offer a comprehensive understanding of potential impacts on the spatio-temporal dynamics of food security in Africa.

The simulation-based approach not only enhances our comprehension of intricate spatio-temporal correlation structures but also equips policymakers with a potent tool for scenario analysis. Policymakers can now evaluate the potential impact of various interventions, spanning from weak to strong spatio-temporal correlation structures. This insight proves invaluable for devising evidence-based policies that effectively address the dynamic challenges of food security in Africa. Given the intrinsic connection between food security, nutritional well-being, and community health, the simulation of diverse scenarios offers a nuanced understanding of potential outcomes. This, in turn, assists public health professionals in designing targeted interventions to enhance the nutritional landscape in the region. Our approach establishes a robust framework for modeling complex spatio-temporal correlation structures.

This paper is structured as follows: In the next section, we describe briefly the methodology of the spatio-temporal interaction models. In Sect. "Simulation study", we describe the Monte Carlo simulation scheme that was used to examine the performance of the four considered models. In particular, we report on goodness of fit (WAIC) and BIAS (RMSE and MAE). The simulation results are presented in Sect. "Results". Finally, in Sect. "Discussion", we summarize our findings and give final remarks.

## Methodology and simulation

### DATA

The Food and Agriculture Organization (FAO) of the United Nations plays a crucial role in supplying data and information to support the attainment of Sustainable Development Goal 2. This goal aims to eradicate hunger, food insecurity, and malnutrition on a global scale. The comprehensive dataset on food and nutrition security can be accessed on the FAO website, where you can also find metadata specifically pertaining to Africa. This metadata comprises variable definitions, data sources, years, and units. To address missing values within the dataset, we employed the missForest, which is random forest algorithm[15]. This research covered a 20-year timeframe, ranging from 2000 to 2019. The study gathered a total of 1080 observations, representing data from all 54 countries on the continent. This dataset encompassed 42 variables, each dedicated to specific aspects of food security and nutrition in accordance with the FAO's definitions. The FAO employs the Food Insecurity Experience Scale (FIES) as a metric for measuring food insecurity. In order to maintain consistency and facilitate comparisons between countries, the FIES Survey Module is implemented among nationally representative samples of the adult population. This standardized approach ensures that data on food insecurity is collected in a uniform manner across different nations [16].

Afridi, Jabbar [16] highlighted the significance of utilizing a convergence of evidence strategy, which involves the use of multiple metrics, to identify the key factors associated with food security and nutrition. The initial set of 40 variables sourced from the FAO dataset underwent Principal Components Analysis (PCA). This method was employed to mitigate potential data loss and tackle the challenge of multicollinearity. It also aimed to overcome limitations associated with the conventional selection of based solely on experts' judgment regarding covariates or factors influencing food security and nutrition. PCA offers a more data-driven and objective approach to identifying relevant covariates (explanatory variables) for food security and nutrition. In the end, a total of ten factors were chosen as explanatory variables in the study. These factors included nutrient intake, average food supply, consumption status, childcare, caloric losses, environment, undernourishment, food or nutritional stability, adequate dietary supply, and newborn feeding practices. Together, these ten factors accounted for approximately 74.6% of the total variance present in the dataset [17].

The results of the Kaiser–Meyer–Olkin and Bartlett's Sphericity test values (with a KMO value of 0.729 and a *p* value of 0.00) indicated that PCA was a suitable approach, as suggested by [18]. Principal component extraction considered eigenvalues larger than one [19]. To ensure that each major component offered distinct information, we applied a VariMax-orthogonal rotation method, in line with Kaiser [19].

The first principal component (PC1) essentially represents nutrient intake and accounts for 19.26% of the total variation in the data. The second principal component (PC2) is associated with factors like food supply, food production, and dietary energy and protein supply. PC2 explains 14.96% of the variation and quantifies the average food supply in Africa. PC3 reflects African consumption levels based on indicators like Gross Domestic Product (GDP) per capita, childhood stunting and overweight, and adult obesity prevalence. It accounts for 10.96% of the variation.PC4, explaining 5.71% of the variance, pertains to childcare factors. PC5 (5.51%) relates to calorie loss, PC6 (4.47%) to environmental influences, PC7 (3.72%) to undernourishment, PC8 (3.43%) to food stability, PC9 (3.39%) to dietary adequacy, and PC10 (3.19%) to infant feeding practices.

The modeling formulation employed in this study is from the spatiotemporal framework. This framework integrates a spatial conditional autoregressive (CAR) prior and an autoregressive (AR) process. By incorporating both spatial and temporal interconnections, this method effectively captures the underlying patterns in the data. The CAR prior is employed to represent the spatial component, taking into account the spatial autocorrelation between neighboring locations. On the other hand, the AR process is utilized to model the temporal component, capturing the temporal autocorrelation over time. By combining these two components, the model can effectively analyze the spatiotemporal dynamics of the data [20].

In Bayesian statistics, when specifying a prior distribution for random variables exhibiting spatial autocorrelation, a common strategy involves combining a uniform prior that encompasses a wide range of values for the intercept (mean) with the intrinsic conditional autoregressive (ICAR) distribution. This approach allows for flexibility and accommodates the spatial autocorrelation structure in the data. Leroux, Lei [21] proposed a remarkable modification to conditional autoregressive (CAR) models that we incorporated into our work. Their suggestion involves using the equation ${\text{Q}}\left({\text{W}}, \rho \right)= \rho \left({{\varvec{W}}}_{{\varvec{d}}} - \mathbf{W}\right)+ \left(1 - \rho \right)\mathbf{I}$, Where, ${{\varvec{W}}}_{{\varvec{d}}}$ represents a diagonal matrix of the weighted average value derived adjacency, and **W** is the n-dimensional adjacency matrix. **I** represent the identity matrix of size$N\times N$, and 1 is an $N\times 1$ vector of ones. The parameter *ρ*, which controls the spatial correlation strength, takes on values between 0 and 1, inclusive. This results in a CAR distribution ${\varphi }_{i}|\rho , {k}^{2},{\varphi }_{j},j\ne i \sim N\left(\frac{\rho \sum_{j=1}^{n}{w}_{ij}{\varphi }_{j}}{\rho \sum_{j=1}^{n}{w}_{ij}+1-\rho } ,\frac{{k}^{2}}{\uprho \sum_{j=1}^{n}{w}_{ij}+1-\rho }\right)$. In this context, ${w}_{ij}$ refers to the element in the nth dimensional adjacency matrix **W** corresponding to the *i*th row and *j*th column. The random effects have a spatial variance of ${k}^{2}$ and autocorrelation of $\rho$

The spatial weight matrix related to the units $i$ and $j$ is represented by each item ${w}_{ij} \in$ **W**. The constituent of ${w}_{ij}$ is ($(i,j)$, which remains the neighborhood matrix with $54\times 54$ dimension. The matrix's nonzero entries reveal whether the two places are neighbors. Typically, the weighted matrix is written as:

$${s}_{ij}=\left\{\begin{array}{c}1\, if\, areas\, i\, and\, j\, are\, neighbours \\ o\, ortherise\end{array}\right.$$

The dataset's spatial autocorrelation is verified by applying Moran's I = $\frac{n}{{s}_{0}}\frac{\sum_{ij}({{w}_{ij}(x}_{i}-\mu )({x}_{j}-\mu ))}{{\sum }_{i}{({x}_{i}-\mu )}^{2}}$, 'n' represents the count of points under investigation,${x}_{i}{x}_{j}$ denote the observed values at two distinct points, $\mu$ denote the expected value of '$x$ and ${w}_{ij}$ signify the spatial weight element. Moran's I range [− 1,1], large values of the relevant metrics are close to other large value clusters when the value is 1, while large values are close to low values when the value of the Moran's I is -1[12, 13]

In the model,${Y}_{it}$ represented the number of severe food insecurity individuals in a country $i$ ($i=\mathrm{1,2},..., 54)$ during year $t(t=\mathrm{1,2},\dots , 20)$ and ${n}_{it}$ denoted the population size of country i at time t. The design matrix **X** represented the food security and nutrition covariates components) are represented by the design matrix ${\varvec{X}}$, and ${\varvec{\beta}}$ was a vector comprising related fixed effects parameters:

1 $${{\text{log}}(\mu }_{it}){=\eta }_{it}={\varvec{X}}\beta +{\text{log}}({n}_{it})$$

By introducing the spatio-temporal random effect ${\psi }_{it}$ to Eq. (1), we obtained the following expression:

2 $${{\text{log}}(\mu }_{it}){=\eta }_{it} {+ v}_{it}$$

To capture the various spatio-temporal features (spatio-temporal interactions) of the multiple aspects of the spatio-temporal random effect ${( v}_{it})$, we decomposed it, leading to the four models in the sequel.

### Spatio-tmporal poisson linear trend model (SPLTM)

3 $${{\text{log}}(\mu }_{it})={\eta }_{it}+{\omega }_{1}+{a}_{i}+({\omega }_{2}+{b}_{i})\frac{t-\overline{t}}{T }$$

where $\overline{t }$ is calculated as $\frac{T+1}{2}$ Here ${\omega }_{1}$ and ${\omega }_{2}$ represent the overall intercept and slope parameters, respectively, and are assigned a flat prior distribution. On the other hand, the incremental trend(slope) and intercept parameters for the ith region, denoted as ${a}_{i}$ and ${b}_{i}$

respectively, are assigned a conditional autoregressive (CAR) prior distribution with different values of* ρ* and ${k}^{2}.$ Specifically, the parameters parameters ${{\varvec{a}}=(a}_{1},\dots ,{a}_{n})$ and ${{\varvec{b}}=(b}_{i},\dots , {b}_{n})$ follow the CAR prior distributions ${\varvec{a}}\sim CAR({\varvec{a}}|{\rho }_{int},{{k}^{2}}_{int},{\varvec{W}})$ and ${\varvec{b}}\sim CAR({\varvec{b}}|{\rho }_{slo},{{k}^{2}}_{slo},{\varvec{W}})$ respectively. Similarly, ${{k}^{2}}_{slo},{{and k}^{2}}_{int}$ represent the variance parameters. The parameters ${\rho }_{slo}{, {\text{and}} \rho }_{int}$ are assigned independent uniform prior distributions in the unit interval (0, 1), indicating that their values can range between 0 and 1. On the other hand, the variance parameters, ${{k}^{2}}_{slo},{{and k}^{2}}_{int}$ follow the inverse gamma prior distribution. In this model, important trends for severe food insecurity are accounted for and their level of significance is accessed.

### Spatio-temporal poisson anova model ( SPAM).

Knorr‐Held [22] proposed a method for analyzing data that incorporates the interaction between space and time by using a model that factors spatial and temporal main effects based on the Analysis of Variance. Equation 4 is derived from Eq. 2 to represent the spatial and temporal effect with attraction.

4 $${v}_{it}={a}_{i}+{b}_{t}+{c}_{it },i=1,\dots , n , t=1,\dots ,T$$

The three sets of parameters indicated by ${a}_{i}$, ${b}_{t}$ and ${c}_{it}$ in Eq. 4 are all considered as random effects, each having its own unique probability distribution.

$${\varvec{a}}|{\rho }_{s},{{k}^{2}}_{s},W \sim CAR\left({\varvec{a}}|{\rho }_{s},{{k}^{2}}_{s},{\varvec{W}}\right),$$

$${\varvec{b}}|{\rho }_{T},{{k}^{2}}_{T},Z \sim CAR\left({\varvec{b}}|{\rho }_{T},{{k}^{2}}_{T},{\varvec{Z}}\right),$$

$${c}_{it }\sim N\left(0,{{k}^{2}}_{I}\right),i=1,\dots ,n, t=1,\dots ,T$$,

Here **Z** is the T × T matrix that represents temporal adjacency, where each element ${z}_{ij}$ takes a value of 1 if the absolute difference between *i* and *j* is equal to 1, and 0 otherwise. It is assumed that the interaction effect ${(c}_{it })$ is independent for all values of i and t.The parameters ${\rho }_{s}$ and ${\rho }_{T}$ are assigned uniform priors with a range of values between 0 and 1, similar to before(Sect. "DATA"), while the variance parameters ${{k}^{2}}_{s}$,${{k}^{2}}_{T}$, and ${{k}^{2}}_{I}$ are assigned inverse gamma priors.

### Spatio-temporal poisson separable model(STSM)

The separable model, which includes an overall time trend with temporal-specific spatial effects, is an alternative model to the one presented in Sect. "Spatio-temporal poisson anova model ( SPAM).". In this case, independent conditional autoregressive (CAR) models are assigned to the spatial effects ${{\varvec{a}}=(a}_{1t},\dots ,{a}_{nt})$ for each $t=1,\dots ,T$ and to **b** = (${{\text{b}}}_{1}$,…, ${{\text{b}}}_{T}$), as shown in the following equation.

5 $${q}_{it}={a}_{it}+{b}_{t}, i=1,\dots , n , t=1, \dots ,T$$

$${{\varvec{a}}}_{t}|{\rho }_{s},{{k}^{2}}_{t},{\varvec{W}} \sim CAR\left({\varvec{a}}|{\rho }_{s},{{k}^{2}}_{t},{\varvec{W}}\right),$$

$${\varvec{b}}|{\rho }_{T},{k}^{2},{\varvec{Z}} \sim CAR\left({\varvec{b}}|{\rho }_{T},{k}^{2},{\varvec{Z}}\right),$$

**Z** refers to the same definition as previously provided in Sect. "Spatio-temporal poisson anova model (SPAM).". The variance parameters ${{k}^{2}}_{t}$, where t ranges from 1 to T, and ${k}^{2}$, are assigned inverse gamma prior distributions, similar to the previous model. The parameters ${\rho }_{s}$ and ${\rho }_{T}$ are assigned independent uniform prior distributions with a range of values between 0 and 1.

### Poisson temporal model for spatio-temporal effect (TMS)

The postulation is made that a particular scenario within the framework of the separable model, as discussed in Sect. "Spatio-temporal poisson separable model(STSM)", corresponds to a temporal autoregressive model with a lag of one. In this special case, the parameter ${b}_{t}$ is equal to zero for all values of t, resulting in the simplification as ${v}_{it}={a}_{it}$, here ${b}_{t}=0$ aimed at the entire $t$ and

$${{\varvec{a}}}_{{\varvec{t}}}\left|{{\varvec{a}}}_{t-1}\right.,{\varvec{W}} \sim N\left(\rho T,{{\varvec{a}}}_{t-1}, {k}^{2}{\varvec{Q}}{\left({\varvec{W}},{\varvec{\rho}}{\varvec{s}}\right)}^{-1}\right), t=2, \dots ., T$$

$${{\varvec{a}}}_{1}\left|{\varvec{W}} \sim N\left(0, {k}^{2}{\varvec{Q}}{\left({\varvec{W}},{\varvec{\rho}}{\varvec{s}}\right)}^{-1}\right),\right.$$

The precision matrix **Q(W,** ${\varvec{\rho}}$ **S)** with spatial dependence is defined in Eq. (1), where the temporal autocorrelation is caused by the mean $\rho T,{{\varvec{a}}}_{t-1}$. The prior distributions are assumed to remain unchanged.

### The chain models

After decomposing the spatio-temporal random effect ${v}_{it}$ and adding it to the linear predictor ${\eta }_{it}$ as shown in Eqs. 2, four different models were obtained: STLTM, SPAM, STSM, and TMS, represented by Eqs. 6–9, respectively:

6 $${{\text{log}}(\mu }_{it})={\eta }_{it}+{\omega }_{1}+{a}_{i}+({\omega }_{2}+{b}_{i})\frac{t-\overline{t}}{T }$$

7 $${{\text{log}}(\mu }_{it})={\eta }_{it}+{a}_{i}+{b}_{t}+{c}_{it}$$

8 $${{\text{log}}(\mu }_{it})={\eta }_{it}+{a}_{it}+{b}_{t}$$

9 $${{\text{log}}(\mu }_{it})={\eta }_{it}+{a}_{it}$$

### Markov chain monte carlo: hamiltonian monte carlo

To construct a Markov chain that converges to the target distribution, Markov Chain Monte Carlo (MCMC) techniques are employed. A Markov chain is constructed by iteratively sampling ${\mathbf{q}}^{(h+1)}$ from the conditional distribution $p\left(\mathbf{q}|{\mathbf{q}}^{h}\right)$ at each time point$h>0$. The process begins with an initial value${\mathbf{q}}^{(0)}$.Importantly, the value ${\mathbf{q}}^{(h+1)}$ at each step depends solely on the current value${\mathbf{q}}^{(k)}$, and is independent of its more distant past values${\mathbf{q}}^{(h-1)}$,${\mathbf{q}}^{(h-2)}$,…, …, ${\mathbf{q}}^{(0)}$ . This specific property, where each step in the chain only depends on the current state and not on previous states beyond the immediate predecessor, is a fundamental requirement for the chain ${\mathbf{q}}^{(h)}$ to be considered a first-order Markov Chain.

The primary challenge in Bayesian modeling convergence lies in constructing a Markov chain that possesses essential properties such as stationarity, irreducibility, aperiodicity, and ergodicity, while also having a stationary distribution equal to the target posterior distribution $\pi \left({\varvec{q}}|{\varvec{y}}\right)$**.** Although there are alternative methods like the Metropolis–Hastings algorithm and the Gibbs sampler, the Hamiltonian Monte Carlo (HMC) is widely regarded as the superior choice. This is because HMC excels in exploring regions of high posterior probability and concurrently employs a Metropolis–Hastings step to address the issue of oversampling peaks while neglecting low probability areas, For a comprehensive understanding of HMC, numerous excellent resources are available, including Lambert [23]. So, executing Hamiltonian Monte Carlo (HMC) for a parameter vector ${\varvec{\theta}}$ requires following the procedures delineated in Lambert's 2018 publication, which are presented in a sequel.

1. Choose an initial location, ${\mathbf{q}}^{(0)}$, randomly from an initial proposal distribution.
2. During iteration *h*, generate an initial momentum (**m**) randomly from a proposal distribution, such as ${\varvec{m}}\sim N({\varvec{\mu}},\Sigma$).
3. Progress the current point${\varvec{q}}$, ***m*** to ${{\varvec{q}}}^{*}$, ${\mathbf{m}}^{*}$ by executing L steps of the leapfrog algorithm, as implemented by the No-U-Turn Sampler (NUTS) [24]. The pair ${{\varvec{q}}}^{*}$, ${\mathbf{m}}^{*}$ represents the updated position of the parameter and its momentum after completing the L leapfrog steps.
4. Compute the Metropolis acceptance probability, denoted as $\alpha ({\varvec{q}},g),$ for the proposal $g=({{\varvec{q}}}^{*}$,${\mathbf{m}}^{*})$ and current point ${\varvec{q}}=\left({\varvec{q}},{\varvec{m}}\right)$ based on the target density $\pi \left({\varvec{q}},{\varvec{m}}|{\varvec{y}}\right)$ where
  $${\text{log}}\pi \left({\varvec{q}},{\varvec{m}}|{\varvec{y}}\right)\propto {\text{log}}\left(\pi \left({\varvec{q}}|{\varvec{y}}\right)\right)+\frac{1}{2}{{\varvec{m}}}{\prime}{\Sigma }^{-1}{\varvec{m}}.$$
5. Generate a random number *u* from a uniform distribution U(0,1), and set ${\mathbf{q}}^{(k+1)}={{\varvec{q}}}^{*}$ if $u<$ $\alpha ({\varvec{q}},g)$ orthewise, set ${\mathbf{q}}^{(h+1)}={\varvec{q}}$

We employed the MCMC algorithm that generates 10,000 samples from the posterior distribution with N = 120,000, burn.in = 20,000, and thin = 10.

## Simulation study

The main aim of our study was to explore how the spatial and temporal correlation structures impact the spatio-temporal dynamics of food security and nutrition in Africa. To achieve this, we conducted a Monte Carlo simulation involving 1,000 random samples (replications) with varying spatial and temporal correlations (0.05, 0.15, 0.25, 0.35, 0.45, 0.55, 0.65, 0.75, 0.85, 0.95) for longitudinal twenty repeated measures in each country. Every aspect of the analysis, which encompassed tasks such as data generation, simulation, and the estimation of model parameters, was executed utilizing the R programming language (R 4.3.1). For each scenario or combination of parameter values, we executed a total of 1000 simulation runs.

**Fig. 1:**
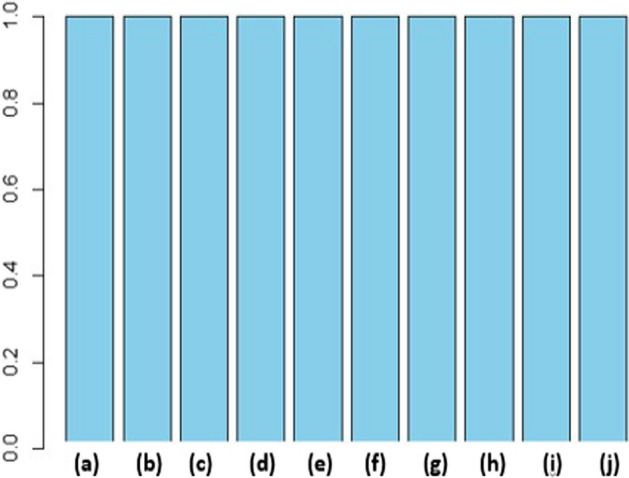
Variable Inflation Factor (VIF) for the 10 explanatory factors of FSN in Africa: **a** nutrient intake; **b** average food supply; **c** consumption status; **d** childcare;** e** caloric losses; **f** environment; **g** undernourishment; **h** food or nutritional stability; **i** adequate dietary supply; **j** newborn feeding practices

The combination of PCA and VIF allows for a more holistic evaluation of multicollinearity, validating the efficacy of the PCA dimensionality reduction while ensuring the dependability of regression model outcomes. This integrated approach fortifies the robustness of statistical analyses, contributing to a more comprehensive understanding of the interrelationships among variables, as evidenced in prior studies [25, 26]. From Fig. 1, we observed that all ten components had a VIF value of 1. The numerical value of 1 for VIF observed for these 10 components confirms that there is no inflation in the percentage of variance (i.e., the standard error squared) for each coefficient. Therefore, the application of PCA is justified.

### Data-generating process

During the data generation process (response variable), we specified four versions of spatio-temporal models to investigate the effects of spatio-temporal correlation using randomly generated (artificial) dependent variables and the 10 explanatory variables (covariates) of food security in Africa identified when the 40 indicators from FAO was subjected to PCA. The spatial units were the 54 countries of Africa. These ten components (covariates) were obtained from the 40 variables provided by the FAO dataset after subjecting them to Principal Component Analysis (PCA) as described in Sect. "Methodology and simulation".

We devised the following iterative algorithm, which was utilized to induce the desired spatio-temporal correlation when generating the values of the dependent variables.

The first version.

- Define the spatial correlation (0.05, 0.15, 0.25, 0.35, 0.45, 0.55, 0.65, 0.75, 0.85, 0.95) and temporal correlation value (0.05, 0.15, 0.25, 0.35, 0.45, 0.55, 0.65, 0.75, 0.85, 0.95) combinations, one hundred pairs.
- Use information in (a) to generate two correlation matrices: the 'spatial correlation matrix' and 'temporal correlation matrix'. These matrices have the specified correlation values and dimensions based on 'num_countries' and 'num_observations', respectively.
- Use the information in (b) to generate the noise using multivariate normal distributions with means of 0 and covariance matrices 'spatial_correlation_matrix' and 'temporal_correlation_matrix', in(b). Here the spatial and temporal data is generated by adding the spatial noise to the coordinates and replicating the temporal noise.
- Use information (c) to calculate a latent variable lamda = exp(spatial datax + temporal data). Where 'spatial datax' represents the x-coordinate of the spatial data for each country. 'temporal data': This represents the temporal noise generated using multivariate normal distributions.
- Generate the observed response variable $y$ where each count is expected to be around the corresponding 'lambda' value. Where $y > 0$
- Fit spatio-temporal interaction model for the four (SPLTM, SPAM, STSM, TMS) models specified in Sect. "The Chain Models"
- Estimate the parameters, and compute the bias and goodness-of-fit measures for evaluation
- Perform steps (a) to (g) iteratively for a total of 1,000 repetitions, each time varying the spatial correlation and temporal correlation values. After each iteration, calculate the relevant parameters accordingly.

For model validation:

I. All other procedures from (a) to (e) remained unchanged, as outlined in the first version.
II. To enable the computation of validation statistics, designate 70 percent of the data for training and 30 percent for testing.
III. Fit spatio-temporal interaction model for the SPAM model
IV. Estimate the parameters, and compute the coverage percentage for validation
V. Perform steps (I) to (IV) iteratively for a total of 1,000 repetitions, each time varying the spatial correlation and temporal correlation values. After each iteration, calculate the relevant parameters accordingly.

### Statistical analyses in simulated datasets

To determine the best predictive model from the four available options, we employed a range of statistical metrics to evaluate and compare their performance. Specifically, we computed the WAIC, RMSE, and MAE for each of these models. The model with the lowest statistical metric values indicates superior predictive ability, striking a balance between model fit and complexity.

#### Goodness of fit test: WAIC

The optimal fit among the four competing models was evaluated using WAIC [27, 28], To calculate WAIC, the log-likelihood of each data point is computed based on the model's parameters. The total log-likelihood is then adjusted for the variance of these log-likelihoods, with a correction term for effective parameters.

10 $${\text{WAIC}}=-2(lpd-pWAIC)$$

Here, "*lpd*" represents the log pointwise predictive density, which is calculated as the summation from i equals 1 to n of the natural logarithm of the density function $p(\left({y}_{i}|\theta \right)$.In this context*,* $p(\left({y}_{i}|\theta \right)$ represents the density of observing the data point ${y}_{i}$ given the model parameters $\theta$. The effective number of parameters, denoted as "pWAIC," is computed as the summation from i equals 1 to n of the variance of the log predictive densities, ${\text{Var}}[{\text{log}}\left\{p\left(\left({y}_{i}|\theta \right)\right\}\right].$ We assessed the goodness of fit by calculating the average model-based WAIC across the 1000 replications.

#### BIAS: RMSE and MAE

Let ${\widehat{y}}_{i}$ denote the estimated severe food security value for the $i$-th observation using a specific method, while ${y}_{i}$ represents the observed value for the same $i$-th observation. The variable n represents the number of data points in each replication. The RMSE is calculated by taking the square root of the average of the squared differences between the actual values and the estimated parameters generated by the model in each iteration. The RMSE can be expressed as follows:

11 $${\text{RMSE}}=\sqrt{\sum_{i-1}^{n}{\frac{\left({y}_{1}-{\widehat{y}}_{i}\right)}{n}}^{2}}$$

The MAE is determined by taking the mean of the absolute differences between the actual values and the estimated parameters obtained from the model in each iteration, as described by Eq. 12.

12 $${\text{MAE}}=\left[\frac{\sum_{i=1}^{n}\left|{y}_{1}-{\widehat{y}}_{i}\right|}{n}\right]$$

Both RMSE and MAE for each replication are arithmetically averaged throughout the 1000 replication.

#### Validation

This involved the validation of the best model among the four competing models. We computed the coverage percentage (CP) for the 95% predictive intervals using the formula ${\text{CP}}=100\frac{1}{{\text{k}}}{\sum }_{{\text{i}}=1}^{{\text{k}}}{\text{I}}\left({{\text{L}}}_{{\text{i}}}\le {{\text{y}}}_{{\text{i}}}\le {{\text{U}}}_{{\text{i}}}\right)$, where ${{\text{y}}}_{{\text{i}}}$ represents the observed value for i = 1,…,k and ${({\text{L}}}_{{\text{i}}},$ ${{\text{U}}}_{{\text{i}}})$ represents the 100(1 − α)% predictive interval for predicting ${{\text{y}}}_{{\text{i}}}$. In this calculation, we employed the indicator function I(·). It's important to note that we used 70% of the data as the training set and the remaining 30% as the test set [29, 30].

## Results

We present the simulation results based on the types of estimated statistical metrics explained in the earlier section. Our primary focus is on evaluating the performance of spatio-temporal models for food security concerning Africa. These metrics are visually represented in a matrix plot and Table.

For the goodness of fit test, it is represented in Table 1. In Table 1 “SP” represents spatial correlation, “TM” denotes temporal correlation, or the correlation over time. The cells in the table are colored-coded to easily identify the best and worst results. Red cells indicate higher or worse values, meaning WAIC. Yellow cells point to lower or better values, signifying less WAIC. The color scheme facilitates interpretation by signaling which areas or spatio-temporal correlation pairs exhibited the strongest or weakest WAIC metric.

**Table 1:**
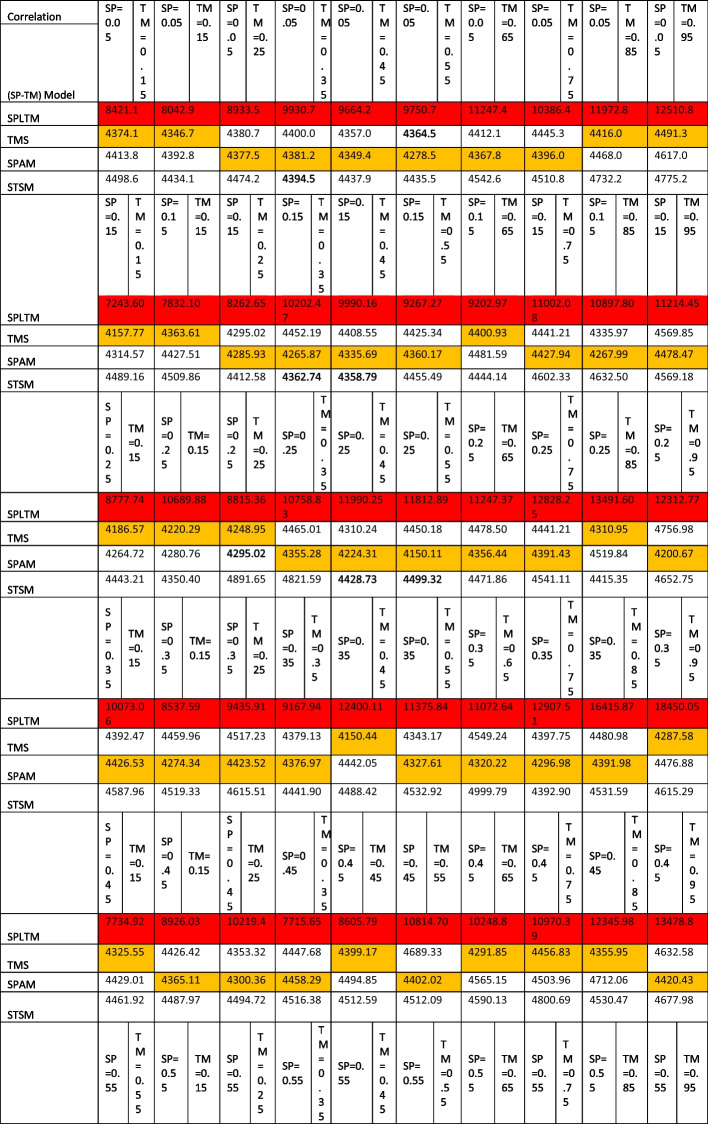

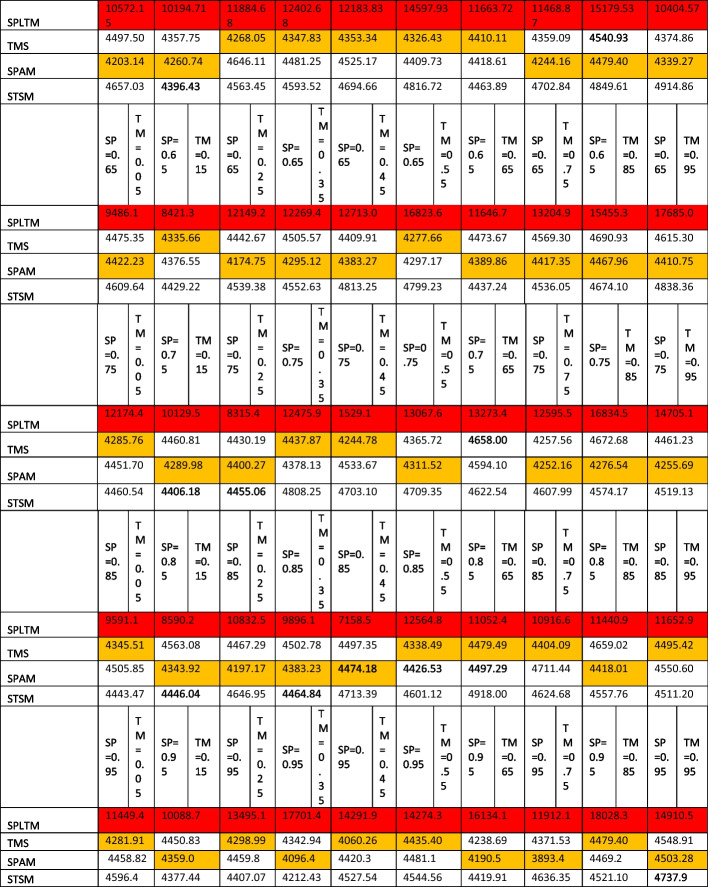
Comparative Goodness of Fit (WAIC) for Spatio-Temporal Models with Varying Spatio-Temporal Correlation

In the table, "SP" represents spatial correlation, and "TM" represents temporal correlation. Cells highlighted in red indicate worse performance, while yellow cells indicate better performance

Matrix plots are a crucial tool for presenting the relationships and patterns discovered in our analysis of the four competing spatio-temporal models. They provided a visual narrative that illustrates how different spatial and temporal correlation structures impact food security and nutrition modeling for Africa. In these matrix plots, the cells are color-coded to represent the values of the parameters, including RMSE, and MAE, for each of the four models under consideration. lower RMSE and MAE values (deeper colors) indicate better model performance. This visualization method helps us to grasp and compare the biases of these models across various spatial and temporal correlation scenarios.

### Goodness of fit assessment

The WAIC analysis provided insights into how sensitive the different models were to the specific combinations of spatial and temporal correlation included in the modelling. Regarding WAIC, Table 1 illustrates that the spatio-temporal Poisson Linear Trend Model (SPLTM) exhibits values ranging from 6000 to 20,000, with the model exhibiting the highest cluster of WAIC values when compared to the four competing models. The highest WAIC value observed was 18,450.05, while the lowest value recorded was 6529.104. Notably, lower WAIC values were observed when both spatial and temporal correlations were weak to moderate, falling within the range of 0.05 to 0.5. This suggests that the model performs better or exhibits more favorable characteristics under these conditions. Conversely, higher WAIC values were observed in scenarios characterized by strong temporal and spatial correlations. This implies that the model's performance does not improve with higher values of the parameters, specifically in cases of elevated spatial and temporal correlation. This behavior indicates that the model is particularly sensitive to increased spatial and temporal correlation, particularly when there are high levels of spatio-temporal correlation.

For spatio-temporal Poisson Anova Model (SPAM), the WAIC values are distributed within the range of 3800 to 5000. The lowest WAIC value observed was 3893.43, while the highest recorded WAIC value was 4917.99 (Table 1). Among the four models compared, SPAM consistently obtained the lowest WAIC scores in the vast majority of simulated scenarios concerning diverse spatial and temporal dependency combinations. This indicates that SPAM excelled in capturing and modeling the underlying spatial and temporal correlations within the data, as determined by the WAIC metric for model selection and performance. In the case of the Poisson Temporal Model (TMS) for the Spatio-temporal correlation effect, as shown in Table 1, the WAIC values also fall within the range of 4000 to 5000 for all 100 pairs of spatio-temporal correlation. The lowest WAIC value was 4060.257, and the highest WAIC value was 4756.981.

It is worth noting an interesting pattern observed in the results: lower WAIC values, indicating better model fit, were seen when the spatial and temporal correlation values included in the simulations ranged from low to moderately strong, specifically when both types of correlations took on values between 0.05 and 0.75. This pattern held for both the SPAM and TMS models. In other words, these two models performed best at capturing the relationships in the data when the spatial and temporal dependencies were in the moderate rather than extremely low or high ranges. This provides useful insight into the conditions under which these types of spatiotemporal models are better able to model correlation structures. WAIC values deteriorated when the data exhibited strong spatio-temporal correlations, as opposed to when these correlations were at lower or moderate levels. This implies that although the SPAM and TMS models exhibited good performance when dealing with modest spatial and temporal dependencies, their capability to effectively capture patterns in the data decreased as the strength of the dependencies across geographical areas and over time increased substantially.

Concerning the performance of the spatio-temporal Poisson Separable Model (STSM), a notable observation is that the performance of the STSM is particularly responsive to variations in both spatial and temporal correlation. The STSM demonstrates relatively strong performance in scenarios characterized by low to moderate levels of spatial and temporal correlation, ranging from 0.05 to 0.55. However, it exhibits higher WAIC values when there is a combination of high spatial correlation with lower temporal correlation. The WAIC values in this case fall within the range of 4100 to 5000, with the highest WAIC value recorded at 4999.791 and the lowest at 4150.11.

### Bias evaluation

**Fig. 2:**
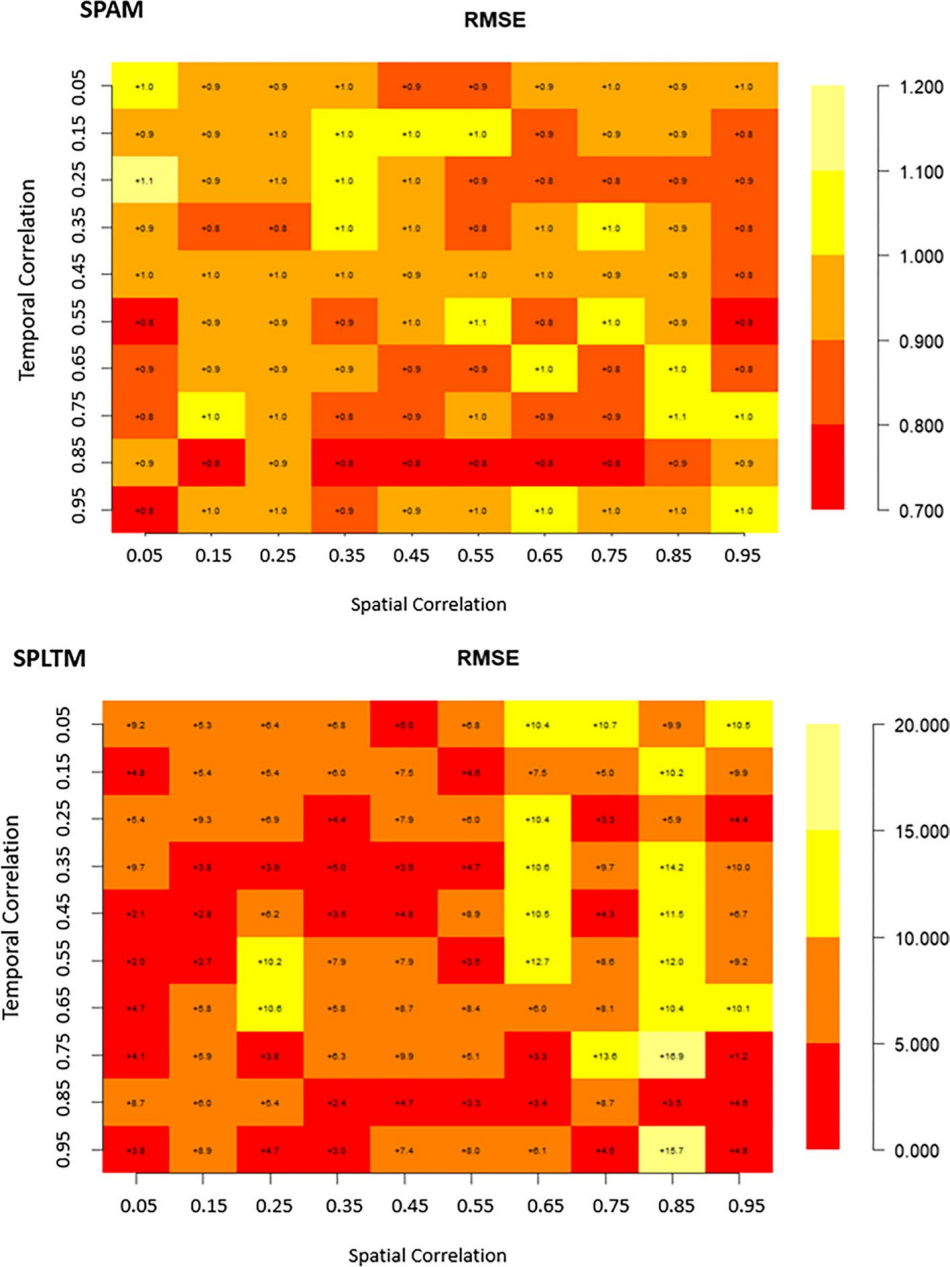

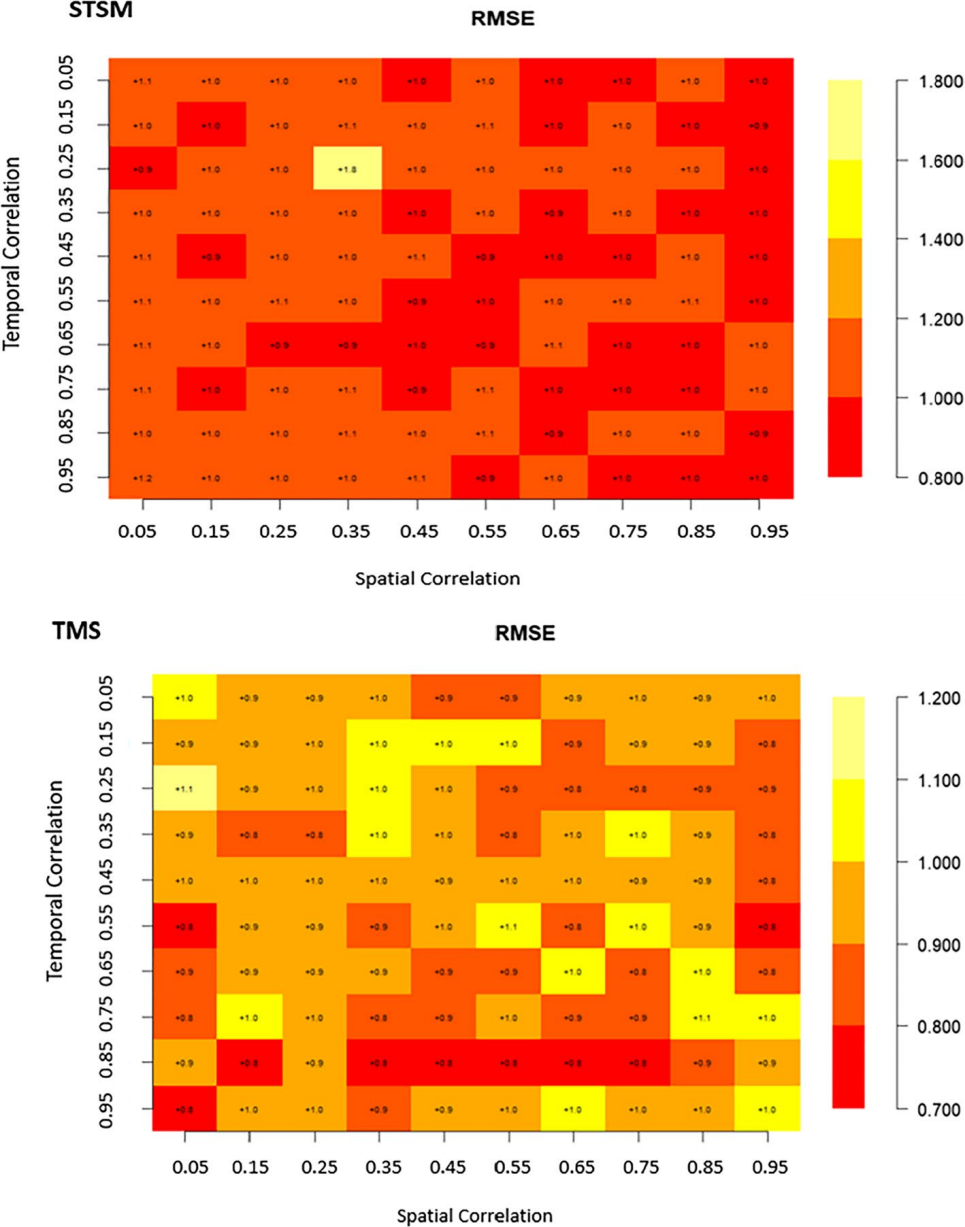

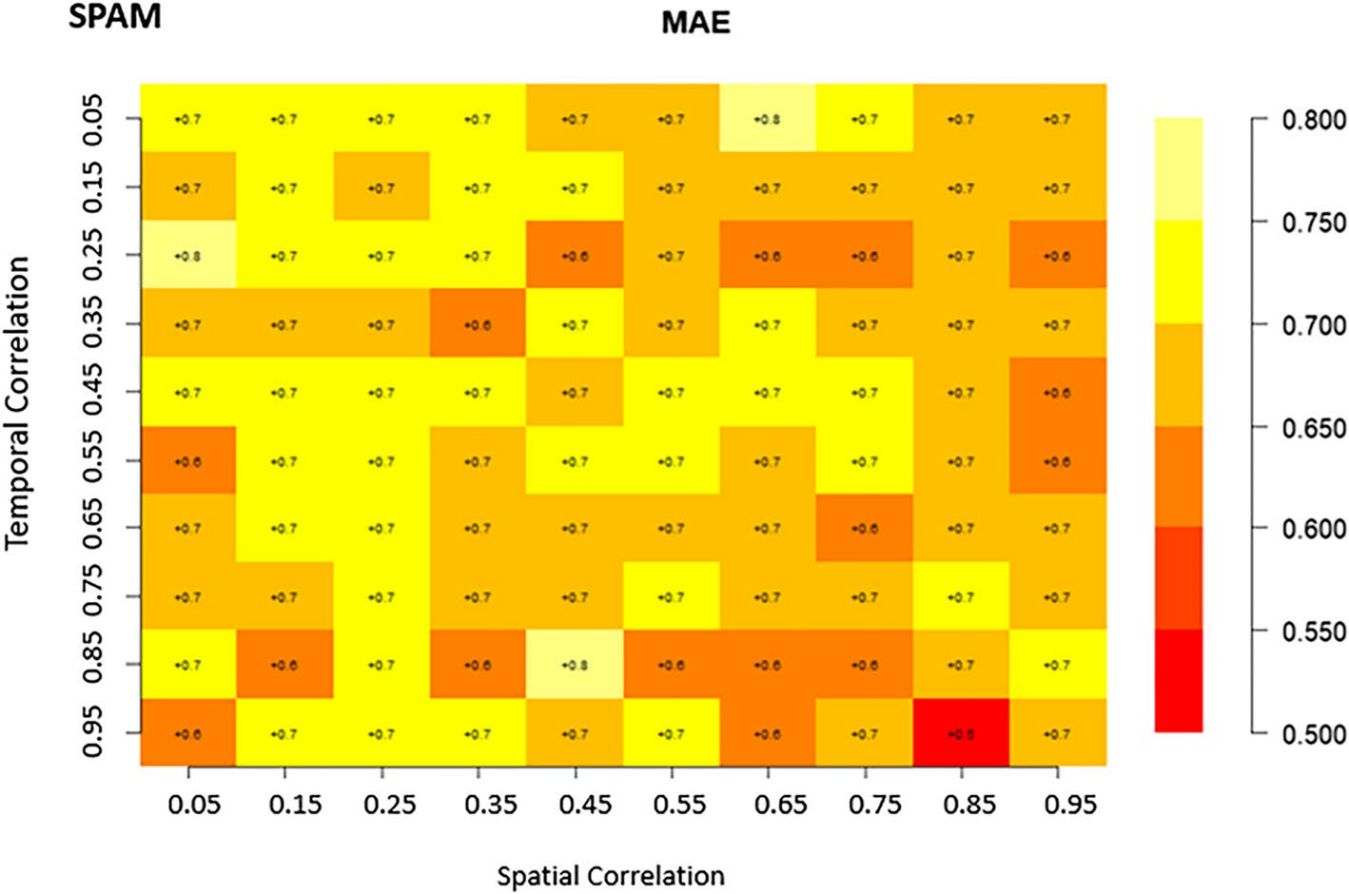
Bias (RMSE) of spatiotemporal models (SPAM, SPLTM, STSM, and TMS) with varying spatio-temporal correlation

Figure 2 depicts the distribution of bias levels, as indicated by RMSE, for all four models. Notably, for both the SPAM and TMS models, the values fall within the range of 0.7 to 1.2, and interestingly, both models exhibited similar distribution patterns. The parameter values vary according to different levels of spatio-temporal correlation, which ranged from 0.7 to 1.00. Notably, there was only one observation exceeding 1.00, occurring when the temporal correlation was 0.25 and the spatial correlation was 0.05. According to Fig. 2, the SPLTM exhibits the highest range of RMSE values, spanning from 1.2 to 16.94. In contrast, the STSM shows a narrower range, with RMSE values ranging from 1.8 to 0.89.

**Fig. 3:**
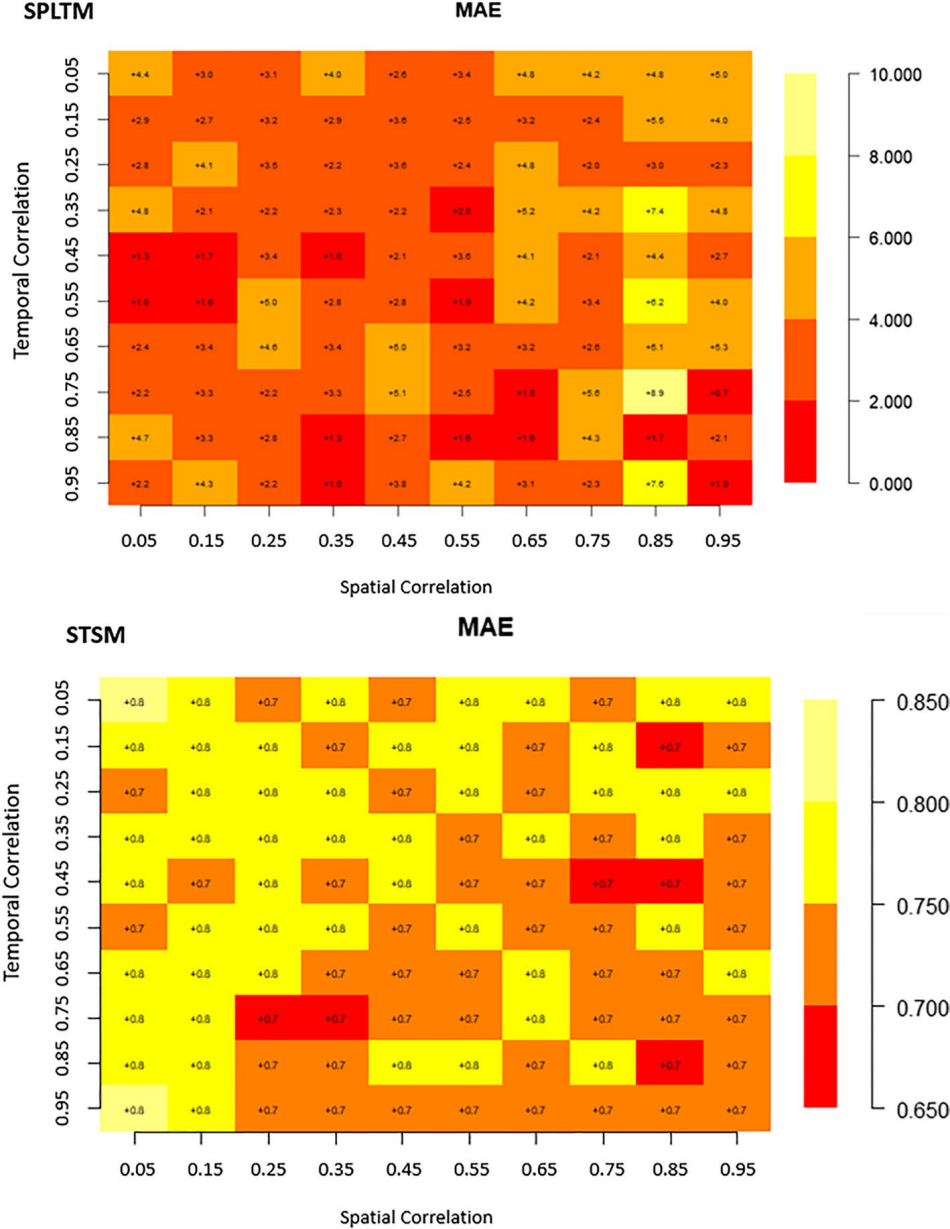

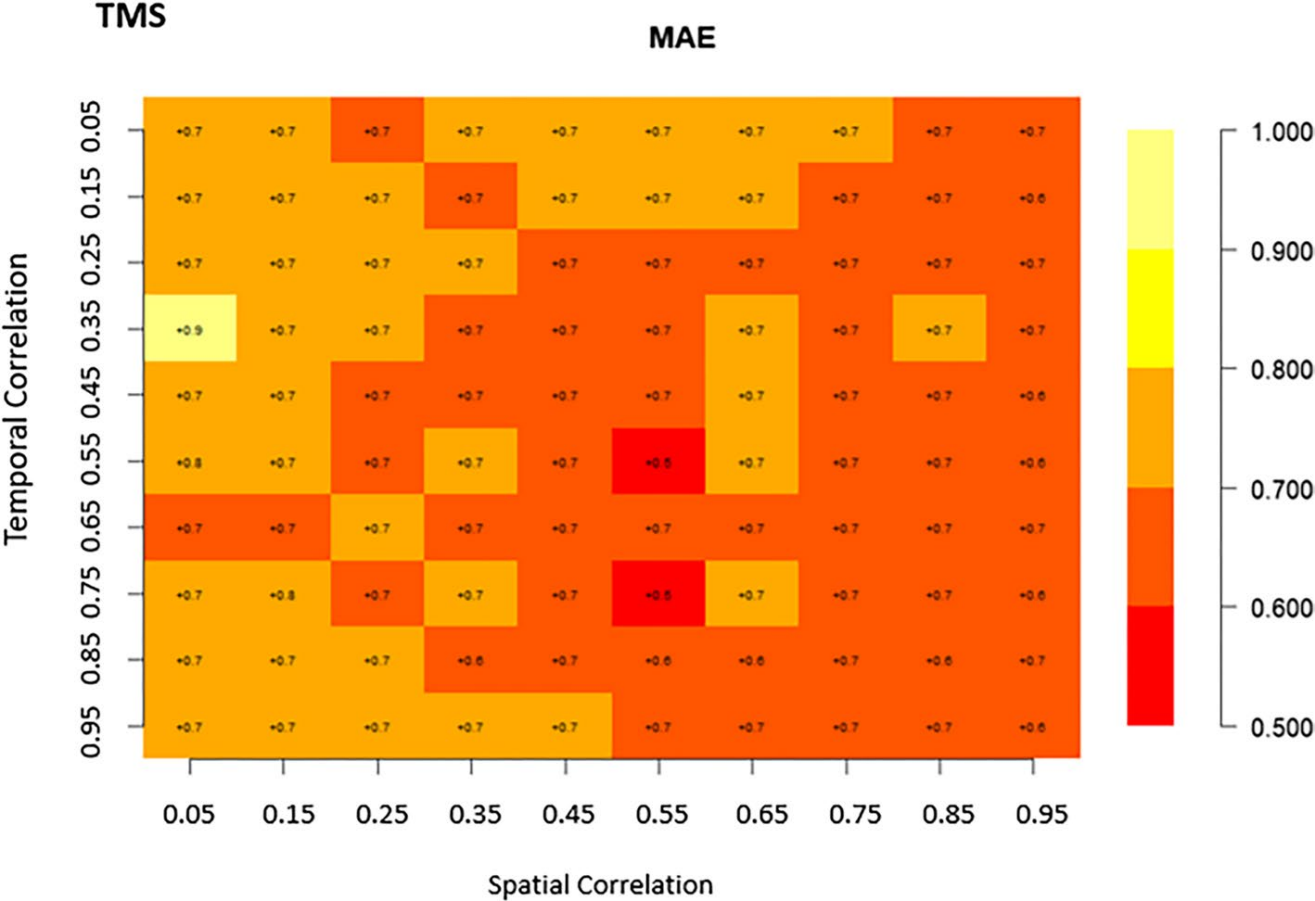
Bias (MAE) of spatiotemporal models(SPAM, SPLTM, STSM, and TMS) with varying spatio-temporal correlation

Regarding the MAE results presented in Fig. 3, the SPLTM model exhibited the widest range of values, spanning from 8.9 to 0.7. In contrast, the SPAM model displayed narrower intervals, with values ranging from 0.8 to 0.5. For the TMS model, the MAE intervals were 0.99 to 0.5 (Fig. 3). Interestingly, the majority of MAE metrics for SPAM fell within the range of 0.6 to 0.75, except in situations when spatial correlation was 0.65 and temporal correlation was 0.05 (Fig. 3). Similar patterns were observed for cases of low spatial (0.05) and temporal (0.25) correlation, as well as spatial (0.45) and temporal (0.85) correlation. These distributions suggest that the SPAM model may not perform well under such conditions, but it excels when there is a strong correlation in both spatial (0.85) and temporal (0.95) (Fig. 3). For the TMS model, lower MAE metrics were clustered when spatial correlation ranged from 0.45 to 0.95 for nearly all temporal correlations (Fig. 3). Regarding the STSM model, the MAE metrics ranged from 0.65 to 0.85, with the highest values observed in scenarios where both spatial and temporal correlations were very weak (0.05) or when temporal correlation was strong (0.95) with weak spatial correlation (0.05) (Fig. 3).

### Coverage percentage-based validation of the selected model: SPAM

**Fig. 4:**
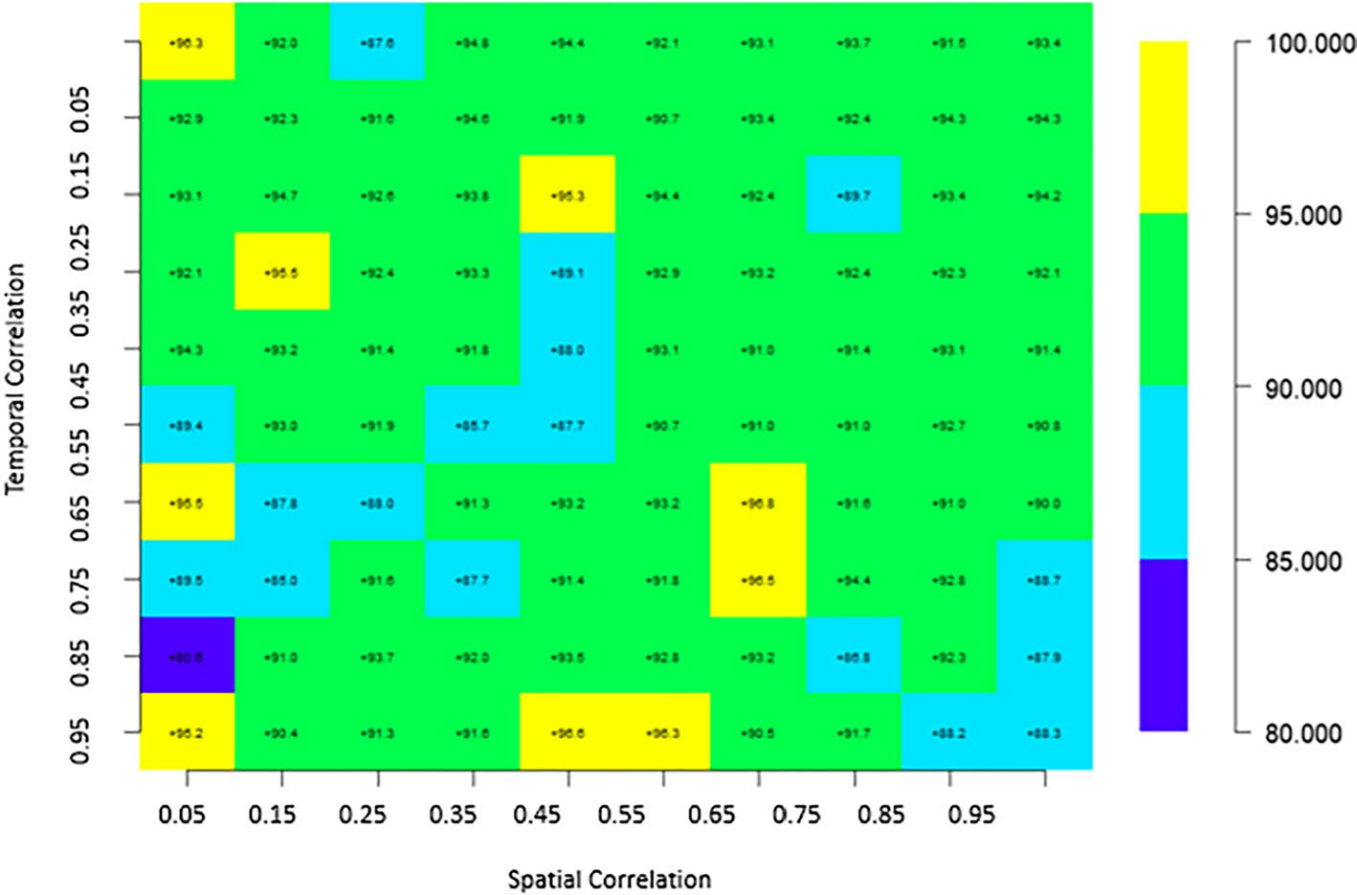
Distribution of coverage percentage for the SPAM model

Based on the principles of statistical learning, the SPAM model is considered the best among the four competing models. Therefore, it is crucial to validate its performance using the 70% train and 30% test split from a machine learning perspective. This validation will help assess the model's capability for handling unseen data across various spatio-temporal correlation scenarios especially concerning Africa’s food security modelling. We noticed a homogeneous distribution of the coverage percentage across almost all variations in spatio-temporal correlation concerning the SPAM. The coverage percentages were noted to vary between 96.8% and 80.6%. Notably, the SPAM model consistently achieved higher coverage percentages (> 90%) for nearly all levels of spatio-temporal correlation, with one exception: when the temporal correlation was high (0.85) and the spatial correlation was weak (0.05) Fig. 4. It achieved its lowest coverage percentage at 80 (Fig. 4), although there were instances (17 observations) where the coverage percentage ranged from 85 to 90.

## Discussion

As we concentrate on evaluating the performance of spatio-temporal models under different spatio-temporal correlation scenarios, we summarize our findings in a table and matrix plots. When necessary, we contextualize our results within the existing literature.

In the table and matrix plot displaying WAIC, RMSE, and MAE values for our four models (SPLTM, SPAM, STSM, and TMS), we have identified intriguing and consistent monotonic patterns that offer valuable insights into their performance and bias characteristics. In essence, these observations underline the sensitivity of all four models to variations in spatio-temporal correlation. On average, each of these models gradually converges towards the true values as spatio-temporal correlation increases, which is reflected by the corresponding increase in WAIC values. These findings strongly support the notion that an augmentation in spatio-temporal correlation levels can indeed impact the performance of these models [31]. Our results reinforced the significance of selecting models that align with the specific patterns evident in the data.

Our study highlights that the SPLTM consistently exhibited WAIC values in these comparisons. This recurrent pattern of SPLTM holding the highest WAIC values raises significant concerns about its suitability for modeling food security and nutrition in the African context. In stark contrast, our findings emphasize the remarkable consistency of the SPAM model. SPAM consistently outperformed the other models, as evidenced by its persistently lower WAIC values when compared to SPLTM, STSM, and TMS for almost all scenarios under study. This robust and unwavering performance strongly suggests that SPAM stands out as exceptionally well-suited for the complex task of modeling the dynamic interplay of food security and nutrition in the African context. The reliability demonstrated by SPAM makes it a top choice for researchers seeking to comprehend and predict the intricate spatio-temporal patterns within this critical domain. These findings align with existing research [13] on the behavior of spatio-temporal interaction models and reinforce the superiority of the SPAM model in this context.

Our analysis of bias using RMSE and MAE unearthed interesting patterns that shed light on the behavior of the four spatio-temporal models. The SPLTM model consistently exhibited relatively larger bias values across a wide spectrum of spatio-temporal correlation values. This consistent pattern of overestimation in food security across diverse spatio-temporal scenarios underscores the model's limitations and its tendency to inflate predictions when necessary. In contrast, the Poisson Temporal Model (TMS) demonstrated relatively more variable bias patterns. In scenarios with low spatial and temporal correlation, it consistently recorded substantial bias values, indicating a potential tendency to overestimate food security. However, in high correlation settings, TMS exhibited a notable shift towards underestimation, showcasing the influence of varying spatio-temporal correlation on its predictive behavior.

The SPAM emerged as the model with minimal bias across most scenarios. This consistent performance highlighted SPAM's reliability in accurately modeling food security dynamics, irrespective of the degree of spatio-temporal correlation. Conversely, the STSM exhibited a notably high bias, consistently underestimating food security. This bias pattern was particularly prominent in areas characterized by low to moderate spatial and temporal correlation, emphasizing the model's limitations in accurately capturing the dynamics of food security in such settings. Our bias evaluation not only highlights the relative performance of these models but also provides researchers with critical insights for model selection based on their specific research questions and the characteristics of their spatio-temporal data. Understanding these bias patterns is paramount to making informed decisions when choosing the most appropriate model for accurate and reliable predictions in the domain of food security.

In light of these findings, we recommend that model selection should be a balanced consideration of both biasness and goodness of fit metrics. A model that exhibits superior goodness of fit but introduces significant bias may not provide accurate predictions, and conversely, a model with low bias but poor goodness of fit may fail to capture underlying spatio-temporal dynamics. Researchers should aim to strike a harmonious equilibrium between these two aspects to select models that align with the specific characteristics of their data and research objectives.

The statistical insights gained from our research have the potential to extend their impact beyond the realm of spatio-temporal modeling. Other disciplines grappling with complex and correlated data, such as epidemiology, environmental science, and economics, can draw upon the methodology and lessons learned in this study. our research fosters interdisciplinary collaboration and opens new avenues for the analysis of intricate spatio-temporal patterns in diverse fields. This cross-disciplinary applicability enhances the relevance and broader impact of our findings.

While the integration of PCA and Bayesian modeling contributes to the efficiency of our methodology, it is crucial to recognize potential computational complexities, particularly with larger datasets. Given that our study focused on 20 temporal observations for each subject (country), future research endeavors could delve into computational optimization techniques. These techniques would be beneficial for handling datasets of varying sizes, ranging from smaller (e.g., 5, 10, 15) to larger (e.g., 25, 30,35) temporal observations. Implementing such strategies would help maintain computational efficiency without compromising model accuracy, addressing scalability concerns associated with different data sizes. The Bayesian modeling approach relies on specific assumptions, and deviations from these assumptions can influence the robustness of the findings. For example, the assumed prior distributions may introduce subjectivity. Future studies could incorporate sensitivity analyses to evaluate the impact of different priors on model outcomes. This would contribute to enhancing the credibility and reliability of our Bayesian approach.

To the best of our understanding, there hasn't been prior research assessing the performance of the four spatio-temporal models (SPLTM, SPAM, STSM, and TMS) in terms of their sensitivity to spatio-temporal correlations using Monte Carlo simulations. It's important to note that, as with any simulation study, our findings are bound by the specific scenarios and data employed in our simulations. Hence, while our analysis of bias and goodness of fit provides valuable insights into the capabilities and constraints of spatio-temporal modeling approaches, it's important to note that these findings might not be directly transferrable to scenarios or contexts that were not explicitly investigated in our study.

## Conclusion

The primary objective of our study was to investigate the influence of spatial and temporal correlation structures on the spatio-temporal patterns of food security and nutrition (FSN) in Africa. We conducted this exploration within the spatio-temporal framework, considering four Bayesian Poisson models: SPLTM, SPAM, STSM, and TSM. Monotonic patterns became evident when we subjected the four competing models to varying spatio-temporal correlations. the SPAM model consistently exhibited the lowest RMSE, MAE, and WAIC values across all spatio-temporal correlations. This notable consistency implies a high level of robustness in the SPAM model's performance. These results strongly suggest that SPAM is a reliable and solid choice for capturing the dynamics of food security. The results of our study provide practical guidance for researchers and practitioners engaged in spatio-temporal modeling. By identifying the strengths and weaknesses of each model under varying degrees of spatio-temporal correlation, we offer a valuable framework for informed model selection. Researchers should strive to find a balanced equilibrium between these two factors (goodness of fit and bias metrics) when choosing models that are in line with their data's unique features and research goals. This knowledge empowers researchers to select models that offer reliability and consistency, enhancing the applicability of their findings.

## Data Availability

The datasets used and/or analysed during the current study available from the corresponding author on reasonable request.

## Abbreviations

SPLTM: Spatio-temporal poisson linear trend model
TMS: Poisson temporal model
SPAM: Spatio-temporal poisson anova model
STSM: Spatio-temporal poisson separable model
WAIC: Watanabe akaike information criterion
RMSE: Root mean square error
MAE: Mean absolute error
MCMC: Markov chain monte carlo
FAO: Food and agriculture organization
PCA: Principal components analysis
PC: Principal component
GDP: Gross domestic product
CAR: Conditional autoregressive
ICAR: Intrinsic conditional autoregressive
HMC: Hamiltonian monte carlo
SP: Spatial correlation
TM: Temporal correlation
